# Deep learning kidney segmentation with very limited training data using a cascaded convolution neural network

**DOI:** 10.1371/journal.pone.0267753

**Published:** 2022-05-09

**Authors:** Junyu Guo, Ayobami Odu, Ivan Pedrosa

**Affiliations:** Department of Radiology, University of Texas Southwestern Medical Center, Dallas, Texas, United States of America; Sun Yat-Sen University, CHINA

## Abstract

**Background:**

Deep learning segmentation requires large datasets with ground truth. Image annotation is time consuming and leads to shortages of ground truth data for clinical imaging. This study is to investigate the feasibility of kidney segmentation using deep learning convolution neural network (CNN) models trained with MR images from only a few subjects.

**Methods:**

A total of 60 subjects from two cohorts were included in this study. The first cohort of 20 subjects from publicly available data was used for training and testing. The second cohort of 40 subjects with renal masses from our institution was used for testing only. A few-shot deep learning approach using 3D augmentation was investigated. T1-weighted images in the first cohort were used for training and testing. Cascaded CNN networks were trained using images from one, three, and six subjects, respectively. Images for the remaining subjects were used for testing. Images in the second cohort were utilized for testing only. Dice and Jaccard coefficients were generated to evaluate the performance of CNN models. Statistical analyses for segmentation metrics among different approaches were performed.

**Results:**

Our approach achieved mean Dice coefficients of 0.85 using a single training subject and 0.91 with six training subjects. Compared to a single Unet, the cascaded network significantly improved the results using a single training subject (Dice, 0.759 vs. 0.835; p<0.001) and three subjects (0.864 vs. 0.893; p = 0.015) in the first cohort, and the results for the second cohort (0.821 vs. 0.873; p = 0.008).

**Conclusion:**

Our few-shot kidney segmentation approach using 3D augmentation achieved a good performance even using a single Unet. Furthermore, the cascaded network significantly improved the performance of segmentation and was superior to a single Unet in certain cases. Our approach provides a promising solution to segmentation in medical imaging when the number of ground truth masks is limited.

## Introduction

Magnetic resonance imaging (MRI) plays a critical role in diagnosis, evaluation, and management of many kidney-related diseases. Furthermore, the role of MRI is increasingly expanding with the extraction of radiomic features from imaging data. Indeed, quantitative assessment of kidney size and morphology or renal mass heterogeneity is possible [1, 2]. Progress in the application of radiomics is however limited by the need to manually segment areas of interest, a time-consuming step [2].

Deep learning, and more specifically convolutional neural networks (CNN), represent state-of-the-art techniques for segmentation in medical imaging [3, 4]. CNN methods extract a complex hierarchy of image features and achieve superior results compared to traditional machine learning methods [4, 5]. CNN segmentation methods for medical images including computed tomography (CT) and MRI were widely used for different organs including brain, heart, and kidney [6–9]. For example, CNN models achieved excellent results for segmenting the kidneys on T2-weighted images of MRI exams of patients with adult polycystic kidney disease [6]. However, construction of such model necessitated the use of 2,000 fully annotated MRI examinations for training and additional 400 fully annotated MRI examinations for testing [6]. In contrast, training a CNN model using a smaller number of datasets (36 MRI examinations) resulted in substantial decrease in the performance of kidney segmentation [10]. These approaches rely on a large amount of data including source images and ground truth masks. Since manual segmentation is considered the reference standard, ground truth is usually obtained after manual delineations of structures of interest by trained personnel (e.g., image analysts, radiologists, etc.). Therefore, creating such masks, particularly in special domains (e.g., MRI), is very costly and time consuming. These challenges can be accentuated for abdominal MRI examinations where ground truth masks have to be drawn for multiple image acquisitions and respiratory motion leads to lack of spatial registration between them.

To overcome the limited availability of annotated datasets, few-shot deep learning, a type of weakly supervised learning, has been proposed [11–13]. With few-shot deep learning, the CNN model is trained from a few datasets containing supervised information resembling the way human brain learns. Few-shot semantic segmentation methods incorporate additional information such as prototype segmentation, object appearances, and human inputs to overcome the challenges resulting from ground truth scarcity [14–16]. Although some proposed few-shot segmentation methods have achieved certain success for evaluation of photographs [17–19], these methods show poor performance on medical images [20]. For the latter, Valverde et al. demonstrated the transferability of the trained CNN segmentation model to new MRI images from different scanners or protocols using one-shot domain adaptation [21]. Chen et al. used one-shot generative adversarial learning to synthesize labeled MR images from CT images to train a CNN segmentation model for MRI bony structure [22]. In addition, Zhao et al. demonstrated one-shot CNN segmentation on MR brain images using a learning-based method for data augmentation, which requires a brain atlas and registration transform [7]. However, implementation of such methods to body MRI segmentation would be impractical as the trained models, CT images, or an anatomy atlas is not available. Alternatively, in this study, 3D augmentation strategies will be applied taking advantage of three-dimensional (3D) MRI data using 3D rotations of the imaging data. Image augmentation is a technique to create more data by altering the existing images for model training to increase its robustness and avoid overfitting. The image augmentation usually includes geometric transformations, kernel filters, random erasing, etc. In addition, cascaded networks can further improve the performance of CNN models [23, 24]. Cascaded networks offer some advantages for medical image segmentation [25–27]. Cascaded architecture combines two separate CNN architectures where the output of the first CNN model is used as an input to the second CNN model to further improve the prediction [28].

Our hypothesis is that deep learning kidney segmentation model can be trained with very limited data by using 3D augmentation. In this study, we investigated the feasibility of kidney segmentation on MR images using CNN models trained with only a few subjects (≤ 6) facilitated by 3D augmentation and a cascaded network structure.

## Materials and methods

### MRI datasets

Two cohorts of subjects were included in this study. The first cohort of subjects was from publicly available data, the Combined (CT-MR) Healthy Abdominal Organ Segmentation (CHAOS) challenge [29]. The CHAOS cohort was used for both training and testing. Axial two dimensional (2D) T1-weighted (T1w) magnetic resonance (MR) images and ground truth kidney masks were downloaded from the CHAOS challenge website [29]. A total of 20 sets of T1w out-phase images and kidney masks from healthy subjects were used to train and test models for few-shot CNN segmentation. The data sets were acquired on a 1.5T Philips MR scanner with a matrix size of 256 x 256/288 x 288, and the number of slices between 26 and 50. The effective slice thicknesses including slice spacing varied between 5.5–9.5 mm. The rest protocol parameters are shown in Table 1. Two of 20 datasets were contrast-enhanced T1w images. This dataset was from publicly available data (https://chaos.grand-challenge.org/) and exempt from Institutional Review oversight.

**Table 1:** Summary of imaging information for all subjects.

Items	CHAOS cohort	RM cohort
Total Number of subjects	20	40
Female/Male	—	22/18
Age (years)	—	22–80
Manufacturer	Philips	Philips, Siemens
Scanner Model	—	Philips: Achieva, Ingenia
		Siemens: Aera, Avanto, Avanto_DOT, Prisma
Field strength	1.5T	1.5T and 3T
TR (ms)	110–255	75–180
TE^†^ (ms)	2.3	2.3 (1.5T), 1.15 (3T) for Philips
		2.37 (1.5T), 1.23 (3T) for Siemens
Flip Angle (degree)	80	55–80
In-plane resolution (mm)	1.44–2.03	0.512–1.953
Slice Thickness/Gap (mm)	5–9 /0.5	5/1
Field of view (mm^2^)	375–520 × 375–520	234–460 × 300–500
Bandwidth (Hz/pixel)	523–744	390–1691

CHAOS = Combined (CT-MR) Healthy Abdominal Organ Segmentation

RM = Renal mass.

—represents unavailable information in the CHAOS website.

† represents the echo time for the first echo; the second echo time is doubled.

The second cohort included 40 subjects who underwent a clinical MRI for evaluation of a renal mass at our institution (RM cohort). The RM cohort were used for testing only. All subjects were imaged in the supine position in 1.5T or 3T MRI scanners from Philips (Intera, Achieva or Ingenia, Philips Healthcare, Best, The Netherlands) or Siemens (Aera, Avanto, Prisma, Siemens Medical Solutions, Erlangen, Germany) from 2016 to 2019. Axial 2D T1w images were acquired using the two-point Dixon gradient echo sequence with the acquisition parameters shown in Table 1. A total of 40 sets of 2D T1w out-phase images were used to further test the models trained using the CHAOS subjects. 36 patients had 1 renal mass, three patients had 2 renal masses, one patient had 3 renal masses. All sizes of renal masses were less than 7 cm. The kidney masks for these 40 datasets were drawn manually by one imaging specialist (1 year experience doing image segmentation/annotation in clinical trials) using 3D Slicer software (https://www.slicer.org/). This retrospective study was approved by the UTSW Institutional Review Board. The need for written informed consent was waived. Imaging information for all subjects in two cohorts is summarized in Table 1.

### Data preprocessing

In this study, T1w MR images are a stack of acquired 2D slices. There is variant intensity in the MR images caused by MR field inhomogeneity. A commonly reported approach (N4 bias field correction) was used for bias field correction [30]. Quantile-based normalization and histogram equalization were used to map intensities of all images into a standard scale between 0 and 255 with better image contrast. Since the slice thickness was larger than the in-plane resolution, interpolation and resampling steps were performed to create an isotropic three-dimensional (3D) dataset (approximately 2×2×2 mm^3^) prior to data augmentation.

### Data augmentation

In this study, augmented transformations included 3D rotation, 3D radial distortion, 2D shear deformation, denoising or adding noise to images, and intensity inversion. Isotropic 3D datasets were rotated in 3D space with different Euler angles for data augmentation. In this study, uniform distributions of Euler axes and angles were used for 3D rotation of MR images. 13 azimuth and 10 polar angles were used to get the axes and 9 uniformly distributed rotation angles for each axis were used to generate 3D rotation transforms. After removing duplicate rotations, a total of 765 3D rotations were selected for data augmentation. 3D rotated exampled images are shown in S1 Fig. Following rotations, one radial distortion and two shear deformations were applied to images with an augmented factor of six. Two kinds of denoising methods were used, smoothing recursive Gaussian filter (sigma of 2) and median filter (radius of 2). Three kinds of noise including additive Gaussian noise (standard deviation: 1% of maximum of image intensity), salt and pepper noise, and Poisson noise (scale factor of 2) were added to MR images. The total number of noised-related augmentations was six. Finally, image intensity inversion was applied to MR images with an additional factor of two. The total number of data augmentation transformations varied from 9,180 to 55,080 depending on the number of selected subjects. To save the training time, all the augmented images were generated in advance. All the preprocessing were performed using python with simpleITK (https://simpleitk.org/).

### CNN model and training

**Fig 1:**
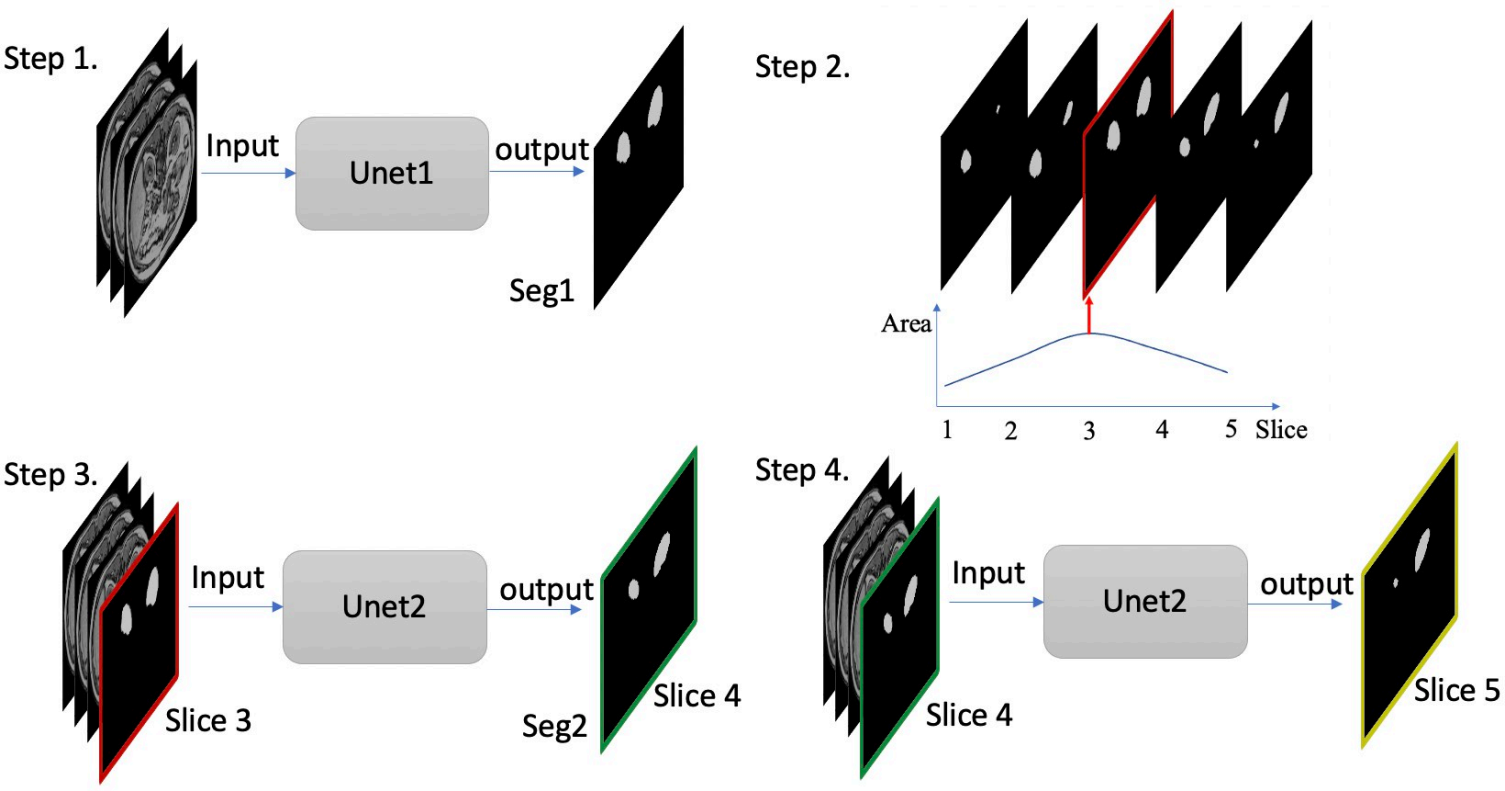
Diagram of the framework of two neural networks including Unet1 and Unet2. The inputs for Unet1 are three consecutive slices in step 1; the best predicted mask (red) was selected based on their area in step 2. The inputs for Unet2 in step 3 are three consecutive slices and the best-predicted mask (red) for the third slice in step 2. Step 4 repeated step 3 for the next slice until covering all the slices. Seg1 indicates the output of the first network; Seg2 indicates the output of the whole network.

The proposed CNN model was a cascaded network including two 2D Unet models shown in Fig 1 [4]. The Unet architecture with a backbone of ResNet34 was used in this study [31]. Unet models based on TensorFlow were downloaded from github (https://github.com/qubvel/segmentation_models). In this study, Unet refers to a Unet architecture with a backbone of ResNet34. The first network (Unet1) was the standard Unet, in which the inputs had three channels composed of three slices. The second network (Unet2) was a slightly modified Unet with four channels composed of three slices and one mask for the third slice. The two Unets were trained independently. The outputs for both networks were the masks for the central slice (i.e., the second slice out of three). In training, the input masks in Unet2 were from ground truth masks. In testing, the masks for all the slices in one subject were predicted first in step 1 (Fig 1). The best predicted mask for the subject was selected in step 2 based on the maximum area of masks, which was usually from the central kidney slice. In step 3, the best predicted mask from Unet1 was used as an input (red) in Unet2 to facilitate the segmentation of its neighboring slice (green). In step 4, this process was repeated for the next slice (yellow) until all masks were predicted for the subject.

**Fig 2:**
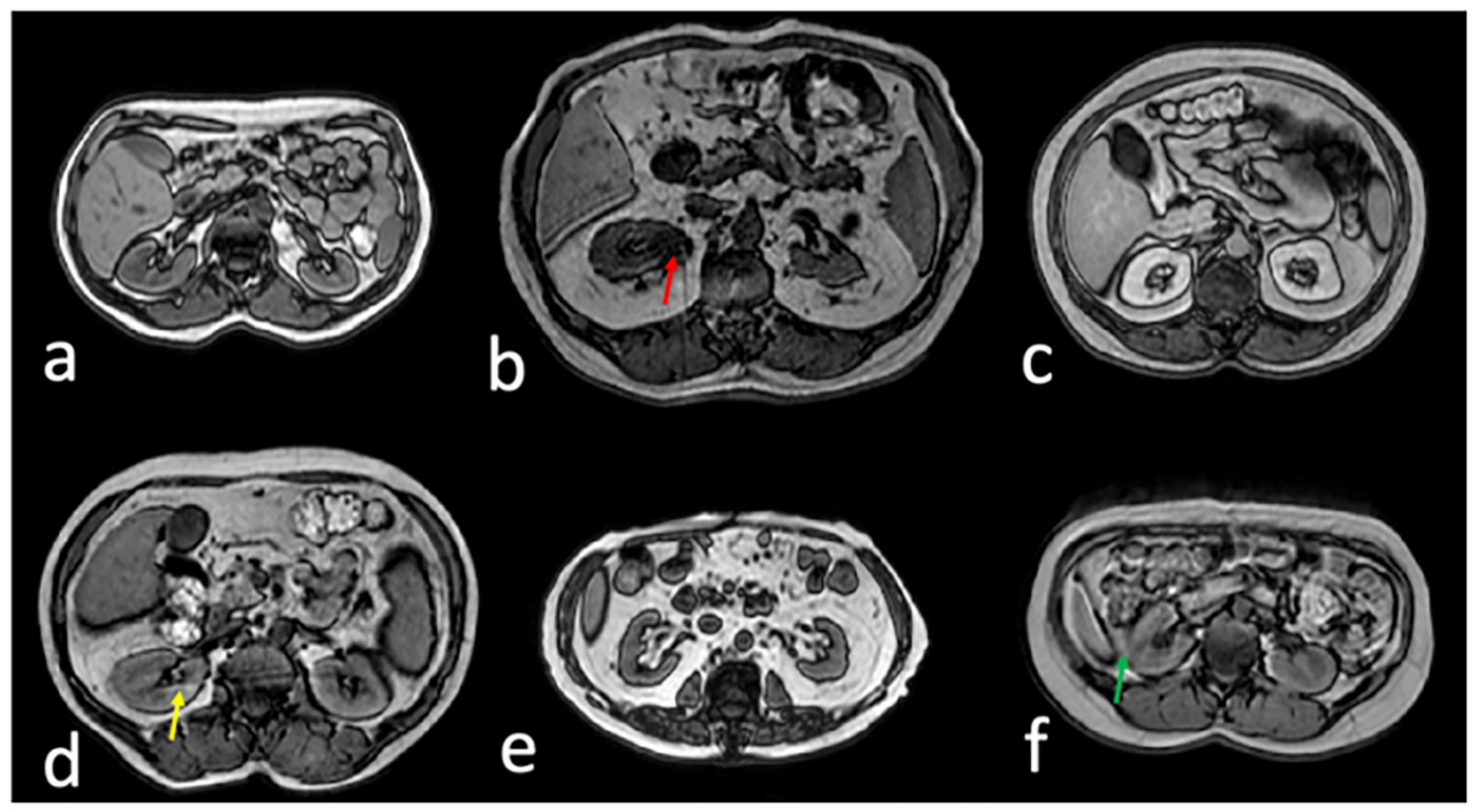
Selected images from six subjects used for training. a. An image from subject 1 with a slice thickness of 5.5 mm; b. An image from the subject 2 with an indeterminate renal lesion in the right kidney (red arrow) and image artifacts with a slice thickness of 6 mm; c. Contrast-enhanced image from the subject 3; d. An image with aliasing artifacts (yellow arrow) from the subject 4; e. An image with different appearance of the kidneys due to abundant hilar fat in renal pelvis from subject 5; f. An image with blurring due to respiratory artifacts (green arrow) from subject 6. The slice thickness for images c-f was 9 mm.

The two Unets were trained with different number of CHAOS subjects and data augmentations in five scenarios: 1. One training subject with a slice thickness of 5.5 mm (Fig 2a) and all noised-related augmentations (N = 55,080); 2. Three training subjects (Fig 2a–2c) with augmentations (N = 18,360) including one Gaussian-noise augmentation; 3. Six training subjects (Fig 2) after randomly selecting half of images from the augmented dataset (N = 9,180). Total number of data augmentations, including the number of subjects, was kept similar (N = 55,080) for the above three scenarios. 4. One training subject (same as in the first scenario, Fig 2a) with fewer augmentations (N = 18,360) including one Gaussian-noise augmentation; 5. One training subject (another subject with a slice thickness of 9 mm, Fig 2f) and the same training setting as in the fourth scenario. The last two trainings were performed to evaluate the effect of the slice thickness on the performance of automatic kidney segmentation. All the data splits between training and testing were performed at the subject level to avoid data leakage.

In addition, to test the reliability and robustness, the trained models using six CHAOS subjects in the above third scenario were further tested using the more heterogenous data in the RM cohort. All the above information was summarized in Table 2.

**Table 2:** Summary of training and testing information for two cohorts.

CHAOS cohort (20 subjects)				
Scenarios	Training		Validation	Testing
	Number of Subjects	Number of Augmentation	Number of Subjects	Number of Subjects
Scenario 1	1	55,080	1*	19
Scenario 2	3	18,360	3*	17
Scenario 3	6	9,180	6*	14
Scenario 4	1^†^	18,360	1*	19
Scenario 5	1^‡^	18,360	1*	19
**RM cohort (40 subjects)**				
—	—			40^††^

† the subject is the same as the one in Scenario 1.

‡ the subject is different from the one in Scenario 1.

* the subjects are the same as ones in the training.

†† Testing used the trained models from Scenario 3.

—not applicable.

Since the kidneys are small objects within the image field of view (FOV) (Fig 2), a loss function, balanced cross entropy (BCE), was used to ameliorate the effect of class imbalance [32]. Balanced cross entropy is defined as follows [33]:

$$Loss=\;-\beta \cdot Y\cdot \text{log}\left(\hat{Y}\right)-\left(1-\beta \right)\cdot Y\cdot \text{log}\left(1-\hat{Y}\right)$$ (1)

Where *Y* is the ground truth mask; $\hat{Y}$ is the predicted mask; *β* is the balancing factor. *β* was set equal to 0.7 for this study.

All the augmented images from the selected subjects (1 to 6 depending on the training scenario used) were randomly split into the training and validation sets (70% for training, 30% for validation). After training, all the images from the remaining subjects (14 to 19) were used for testing all the trained segmentation networks.

All the trainings were performed on the high-performance computing (HPC) cluster (one node with one Nvidia v100 GPU, 32G memory). The total data size of the augmented images including six training subjects was about 2.1 terabytes (TB). The hyperparameters including a batch size of 100 and the epoch number of 5 were used for the first Unet (Unet1); a batch size of 50 was used for the second Unet (Unet2) due to the limitation of GPU memory. The best weights were saved for the prediction. The training time for each CNN model varies from a few days to two weeks depending on the amount of data used for the training.

### Evaluation of kidney segmentation

The best models were used to predict kidney masks of the testing dataset. For the first Unet, the two different types of prediction, direct vs. augmented, were generated. In the direct prediction, the pre-processing steps in the prediction stage were the same as those steps in the training stage. In the augmented prediction, MRI images after the aforementioned pre-processing steps were further adjusted to nine different window levels for prediction. The predicted mask with the maximum area was selected for further processing. After all the masks were generated, one additional post-processing step was performed to extract the largest connected component following the projection from 3D images to 1D signal along the slice direction. This step eliminated the overestimated parts outside the kidneys and further improved the results.

Segmentation metrics, Dice and Jaccard coefficients (a.k.a. Intersection over Union (IoU)), were calculated to compare between the ground truth masks and the predicted masks in the different trainings. Dice and Jaccard coefficients were defined as follows [6]:

$$Dice=\frac{2TP}{2TP+FP+FN}$$ (2)

$$Jaccard=\frac{TP}{TP+FP+FN}$$ (3)

Where TP is true positives; FP is false positives; FN is false negatives.

Statistical analyses, paired and unpaired T-Tests, for segmentation metrics among different approaches were performed using python with SciPy. A P value <0.05 was considered significant.

## Results

All the results were grouped and presented for different considerations such as the number of training subjects, types of prediction, and different network models. For convenience of description, Seg1 refers to the results for the first Unet; Seg2 refers to the results for the whole cascaded network in Fig 1.

**Fig 3:**
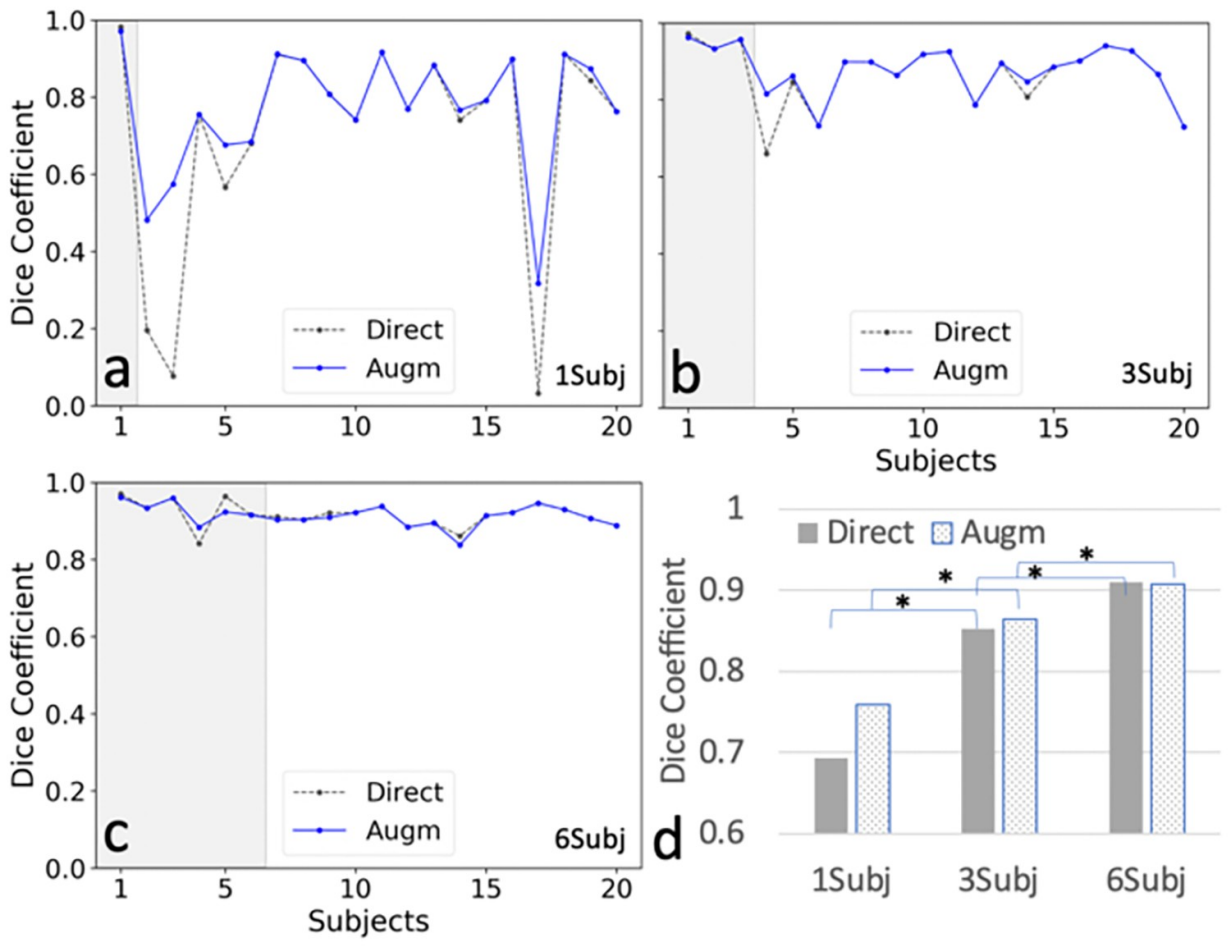
Dice coefficient plots for all subjects using the first deep neural network (Unet1) and two methods, direct vs. augmented (Augm), in the prediction stage. a. Results from a model trained using subject 1 (1Subj); b. Results using subjects 1,2, and 3 (3Subj); c. Results using the subjects 1–6 (6Subj). d.) Bar plots illustrate comparative dice coefficients for the models used in a, b, and c above. Although the training data were from different number of subjects, the total number of images for training was kept the similar using data augmentation. Direct indicates the results predicted directly using the trained model; Augm indicates the results predicted after the images are augmented by adjusting window levels in the prediction stage. Gray areas in a-c indicate the training data sets. * represents significant difference using unpaired T-Tests. P values from the left to the right are 0.031, 0.016, 0.015, and 0.027, respectively.

Fig 3 (Scenario 1–3) shows the plots of Dice coefficients of Seg1 prediction of renal segmentation using the first Unet network and different number of training subjects in the CHAOS cohort. Fig 3a (Scenario 1) shows the results from the model trained using only a single subject (Fig 2a). Dice coefficients were close to 0.8 for most of 19 testing subjects except for a few subjects with dramatically different image contrast (e.g., subjects 3 in Fig 2c and subject 17 with contrast-enhanced T1w images). However, the segmentation results in Fig 3b (Scenario 2) were dramatically improved by adding two more subjects into the training cohort: one subject with a large amount of perirenal fat and a kidney lesion (indeterminate based on T1w alone, Fig 2b) and another subject with contrast-enhanced images (Fig 2c). Fig 3c (Scenario 3) shows that the segmentation results were further improved by adding three additional subjects (Fig 2d–2f) into the training set.

The summarized results using different number of subjects are shown in Fig 3d. The mean Dice coefficients for all the testing subjects reached a value of 0.91 for seg1. In addition, Fig 3 shows that Dice coefficients using different number of training subjects were significantly different in all cases. Dice coefficients using the augmented prediction were substantially larger than Dice coefficients using the direct prediction for some subjects. However, Dice coefficients in the two predictions were not significantly different. The difference of Dice coefficients was reduced as the number of training subjects increased.

**Fig 4:**
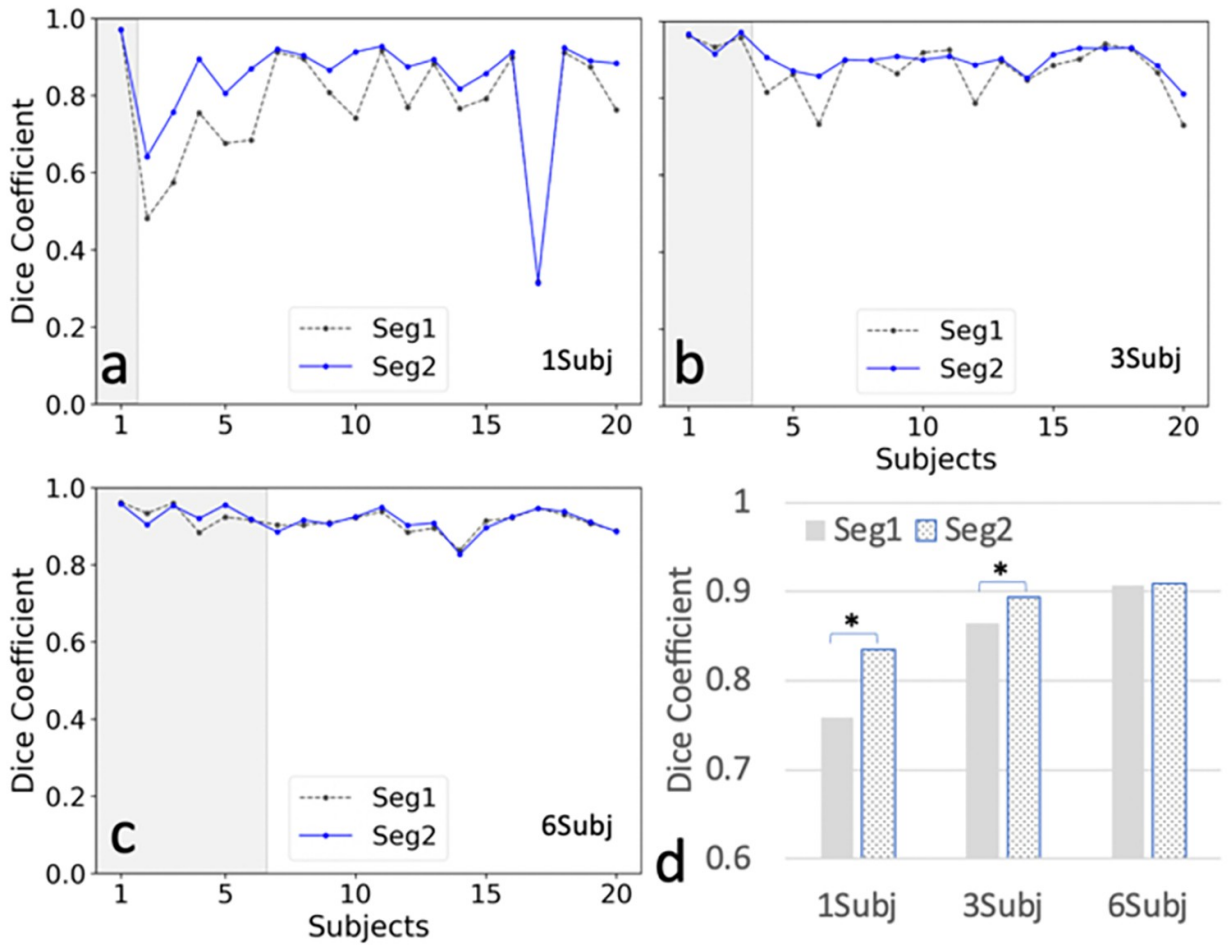
Comparison between two results (Seg1 vs. Seg2) of Dice coefficients using the different trained models. a. Dice coefficient plots from a model trained using subject 1 (1Subj); b. Dice coefficients of model using subjects 1, 2, and 3 (3Subj); c. Dice coefficients of a model using subjects 1–6 (6Subj). d. Bar plots illustrate comparative dice coefficients for the models a, b, and c above. Although the training data were from different number of subjects, the total number of images for training was kept the similar using data augmentation. Seg1 indicates the results predicted using the first network alone; Seg2 indicates the results by using the cascaded network (including two Unets) in Fig 1. Gray areas in a-c indicate the training data sets. * represents significant difference using paired T-Tests. P values for 1Subj and 3Subj are <0.001 and 0.015, respectively.

**Fig 5:**
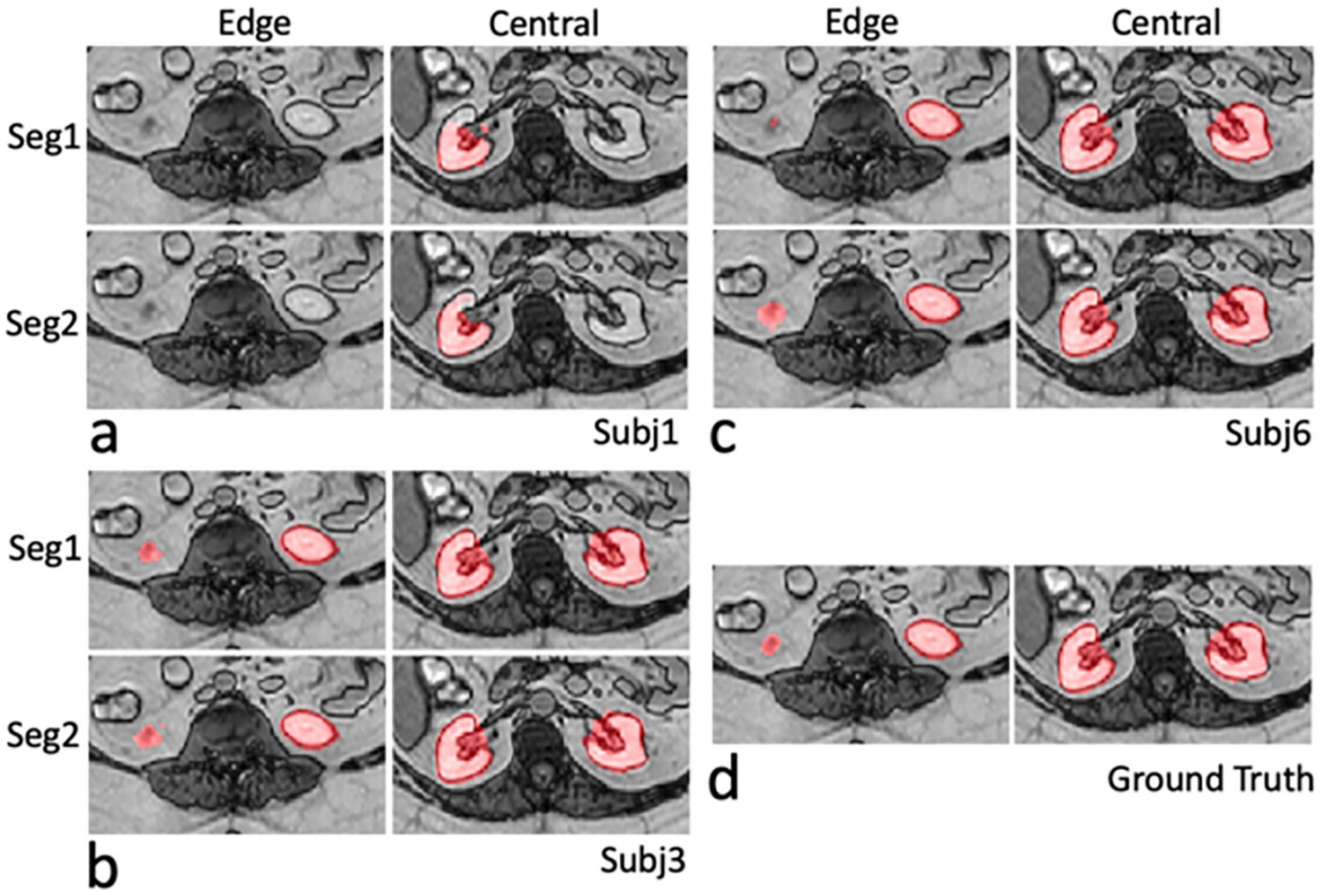
Comparison between two results (Seg1 and Seg2) for two selected images (i.e., at the level of the lower pole and hilum of the kidney) from subject 17 in Fig 4a using different number of training subjects. a. overlaid images using one training subject; b. overlaid images using three training subjects; c. overlaid images using six training subjects; d. overlaid images with ground-truth masks. Subject 17 represents the subject with the lowest Dice coefficient in Fig 4a. Images were cropped due to the limited space. Red regions represent predicted masks or ground truth masks. Seg1 indicates the results predicted using the first network alone; Seg2 indicates the results by using the cascaded network (including two Unets) in Fig 1.

Fig 4 (Scenario 1–3) shows the plots of Dice coefficients of Seg1 and Seg2 using different number of training subjects in the CHAOS cohort. Dice coefficients of Seg2 were significantly higher than Dice coefficients of Seg1 when using one and three training subjects (p<0.001 and p = 0.015, respectively). The cascaded network (Seg2) shows a clear advantage over a single Unet (Seg1) in the case using a single training subject. This advantage disappeared when using six training subjects. Table 3 summarizes the Dice and Jaccard coefficients for the above analyses. Fig 5 shows representative examples of predicted masks of two selected slices (i.e., at the level of the lower pole and hilum of the kidney) for subject 17 with the lowest Dice coefficient trained using one subject in Fig 4. When trained using only one subject, both models (Seg1 and Seg2) failed to predict kidney masks on both the lower pole and hilum slices (Fig 5a). In contrast, both models successfully predicted the kidney masks when trained using three or six subjects (Fig 5b and 5c).

**Table 3:** Summary metrics for different models.

Metrics: m ± SD [min/max]		Seg1		Seg2
		Direct	Augmented	Augmented
Dice	1 Subject	0.693±0.272 [0.032/0.916]	0.759±0.156 [0.318/0.916]	0.835±0.140 [0.314/0.928]
	3 Subjects	0.852±0.079 [0.660/0.941]	0.864±0.062 [0.730/0.941]	0.893±0.030 [0.811/0.931]
	6 Subjects	0.910±0.022 [0.861/0.946]	0.907±0.026 [0.838/0.946]	0.910±0.022 [0.827/0.949]
Jaccard	1 Subject	0.584±0.258 [0.016/0.845]	0.634±0.181 [0.189/0.845]	0.736±0.160 [0.186/0.865]
	3 Subjects	0.750±0.113 [0.493/0.888]	0.766±0.092 [0.575/0.888]	0.809±0.048 [0.682/0.871]
	6 Subjects	0.836±0.036 [0.756/0.898]	0.831±0.042 [0.721/0.898]	0.834±0.049 [0.706/0.903]

Shown are the results for the first Unet (Seg1) and the whole cascaded network (Seg2).

Direct indicates the results predicted directly using the trained model; Augmented indicates the results predicted after the test images are augmented by adjusting window levels. m: mean value; SD: standard deviation.

**Fig 6:**
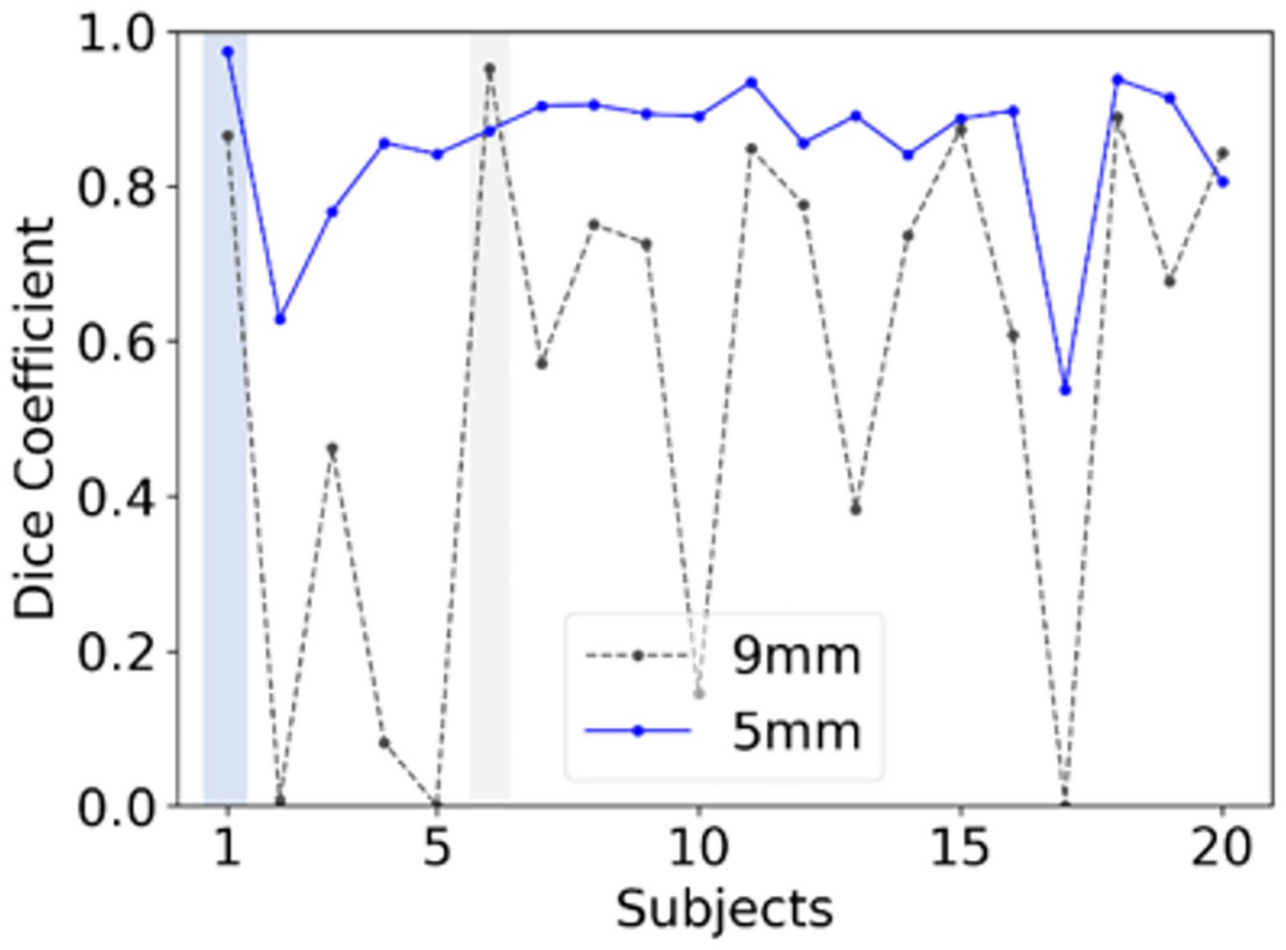
Comparison of predicted Dice coefficients between two single-subject models (Seg1) trained using two different subjects (slice thickness: 5mm vs. 9mm). The dash line represents the results predicted using the model trained using the images of subject 1 with a slice thickness of 5.5mm; the solid blue line for subject 6 with a slice thickness of 9 mm. Gray and light blue bars indicate the training data sets in each training (Gray for 9mm. Light blue for 5mm).

Fig 6 (Scenario 4,5) shows the plots of Dice coefficients of Seg1 for two different single-subject trainings in the CHAOS cohort, in which two subjects had different slice thicknesses (5.5 mm vs. 9 mm). The results were from the first Unet using the augmented prediction. The mean Dice coefficients with standard deviations were 0.85 ± 0.10 for the training with 5mm (subject 1) and 0.56 ± 0.33 for the training with 9 mm (subject 6).

**Fig 7:**
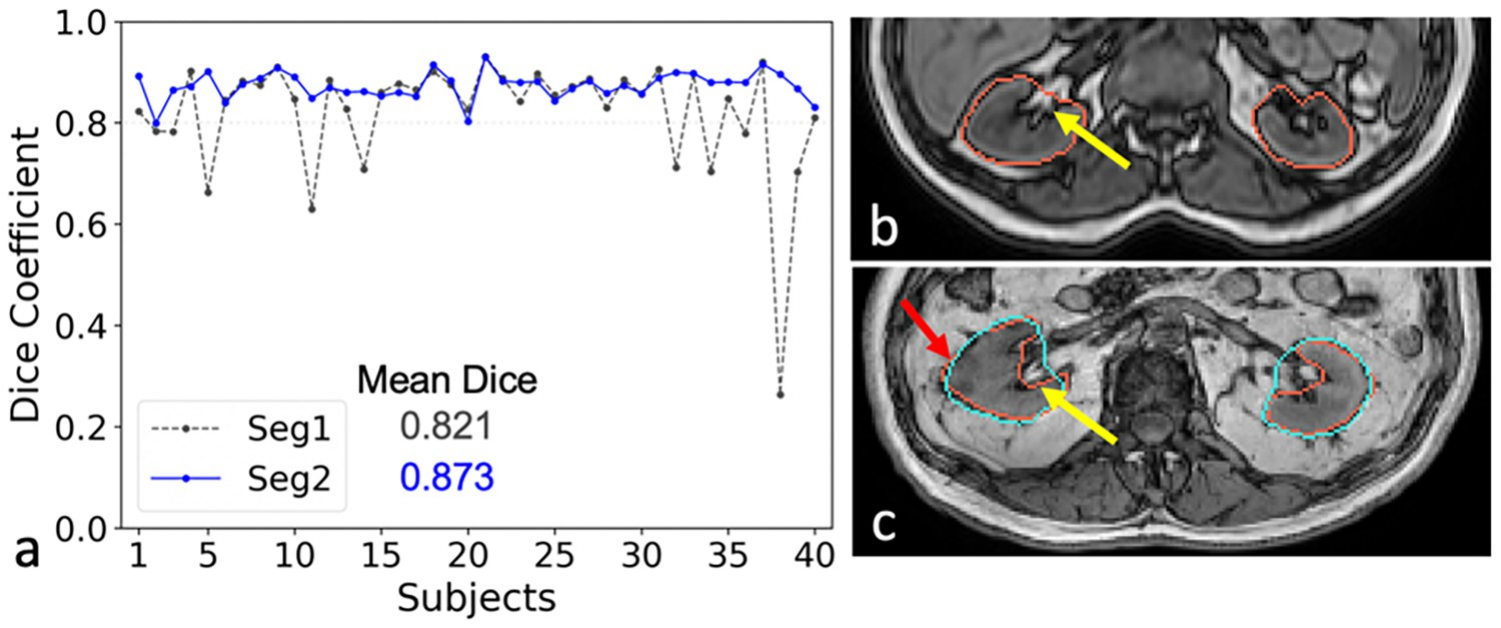
Testing results from renal mass (RM) cohort. a. Dice coefficient plots for 40 RM subjects predicted by a model trained using six CHAOS subjects; b. The ground truth masks in CHAOS (red line) included the renal hilar fat (yellow arrow) (Fig 2a); c. The ground truth masks in the RM cohort (red line) did not include the renal hilar fat (yellow arrow). However, the predicted mask from the Seg2 network (light blue line) in this representative RM subject included the hilar fat mass due to training with CHAOS data. The discrepancy in the annotation of ground truth images between the training dataset (CHAOS cohort) and the testing dataset (RM cohort) resulted in a lower dice coefficient of 0.869 for this subject. The red arrow points to a small renal mass.

Fig 7 shows the testing results of 40 RM subjects predicted using the model trained using six CHAOS subjects. Fig 7a shows the plots of Dice coefficient of Seg1 and Seg2 for 40 RM subjects. The mean values of dice coefficients for the Seg1 and Seg2 networks were 0.821 and 0.873, respectively. The standard deviations for Seg1 and Seg2 were 0.115 and 0.027, respectively. Dice coefficients of Seg2 were significantly higher than that of Seg1 (p = 0.008) using a paired T-Test. The cascaded network (Seg2) shows a clear advantage over a single Unet (Seg1) in the RM cohort. Differences between the predicted masks and the ground truth were explained by inclusion of hilar fat in the central kidney, which is consistent with the ground truth masks in training CHAOS data (Fig 7b). However, the hilar fat was excluded during manual annotation of the ground truth masks in the RM cohort (Fig 7c).

## Discussion

Our few-shot CNN approach obtained high accuracy for kidney segmentation with a mean Dice coefficient of 0.91 for the CHAOS cohort using six training subjects. The mean Dice coefficient for the CHAOS cohort reached a value of 0.85 using a single training subject, which compares favorably to a previous reported Dice value of 0.78 for kidney trained using 36 subjects [10]. Our best mean Dice coefficient of 0.91 was close to reported Dice coefficient of 0.96 for kidney segmentation with a model trained using 2000 subjects [6]. Similarly, our results are comparable to the best Dice coefficient of 0.95 reported for a model trained using all 20 subjects in task 5 of the CHAOS challenge for MRI segmentation of liver, kidney and spleen [29].

Feng et al. used interactive few-shot learning to achieve a Dice coefficient of 0.58 for kidney segmentation trained using four CT datasets [20]. Cui at al. achieved a dice coefficient of 0.77 for kidney segmentation on CT images using one-shot segmentation based on distance metric learning [34]. Although kidney segmentation may be more challenging on MR images than CT images due to lower spatial resolution of MRI (i.e., blurrier boundaries) and more common image artifacts, we achieved a higher Dice coefficient, for MRI kidney segmentation, of 0.85 using one-shot training and 0.91 using six-subject training.

In the more heterogeneous RM cohort, our approach achieved a mean Dice coefficient of 0.873 using the model trained using six CHAOS subjects despite the inconsistency of the ground truth (i.e., inclusion/exclusion of renal hilar fat). Compared with the CHAOS data acquired on a single MRI scanner, the RM data were acquired on six different MR scanners from different vendors, field strength, and greater variability of acquisition parameters (Table 1). Further improvements in segmentation accuracy, as reflected by the Dice coefficients, are expected with standardization of the image annotation of ground truth datasets.

A major challenge for deep learning models trained using a large dataset is their poor generalizability across institutions, MRI scanners from different vendors, or different MR sequences. One CNN segmentation model trained in one institution may fail using similar data from another institution. Moreover, a CNN segmentation model trained for T1-weighted images may fail for T2-weighted images. Indeed, the general thought is that CNN models should be retrained for each new dataset available. However, it is impractical to recreate ground truth masks for each new large dataset due to unacceptable high cost and time requirements. Our results support the idea of using few-shot CNN for training a new model or transferring a trained model to a new dataset, different than the one originally used to train the model. In this study, we demonstrate that it is feasible to train CNN models for kidney segmentation with high accuracy using only a few subjects. The selection of subjects in the training set was performed to enable inclusion of a variety of MR images with different contrast, the presence of artifacts, or lesions (Fig 2) to reduce underfitting in a trained model. The images from the rest subjects were used for testing. In addition, we demonstrate that the trained model using six CHAOS subjects was very reliable and robust in the model testing using a larger RM cohort with 40 subjects (Fig 7a).

The cascaded network (Seg2) was more stable and consistent showing a clear advantage over a single Unet (Seg1) for the larger RM cohort (Fig 7a). The performance of Seg1 varied for different subjects due to residual field inhomogeneity and different image artifacts in Fig 4. The performance of Seg1 may be superior for central kidney slices compared to those slices located near the kidney poles in one subject. The best predicted mask of one central slice from Seg1 was selected to facilitate the segmentation of its neighboring slice to achieve better results in the second Unet (Fig 1). However, the improvement using the second Unet may be limited when Seg1 already got favorable outcomes or got very poor outcomes (Fig 4).

The challenge of underfitting is demonstrated in the results from the model trained using one subject. Underfitting occurs when the training images from a single subject don’t have enough complexity to represent the images from other subjects correctly. The model trained using one subject performed well only for certain subjects with images resembling those of the training subject; but performed poorly for the other subjects with different image contrast (Fig 3a). Although the proposed augmented prediction partially ameliorated this problem in comparison with the direct prediction, evidence of underfitting remains apparent in those subjects with different image contrast in Fig 3a. Furthermore, the cascaded network further diminished the underfitting problem, as shown in Fig 4a. In contrast, by including a few subjects with different image contrast in the training set, we observed a dramatic improvement in the performance of the trained model. Indeed, underfitting was greatly reduced and virtually non-existent when more subjects with different image contrasts were added to the training set (Figs 3 and 4).

Data augmentation increases the number of training datasets by applying a set of transformation to the images and masks [35] Data augmentation improves the reported performance of models, especially when training using small datasets [36]. In this study, we exploited 3D features of MR datasets in data augmentation. Specifically, our 3D rotation augmentation strategy required interpolation and resampling procedures. As a result, aliasing artifacts arising from these procedures became more serious as slice thickness increased [37–39]. Although the axial images may appear somewhat similar, the coronal reconstruction for Subject 6 (i.e., using 9 mm axial slices) had more profound aliasing and blurring artifacts than the coronal reconstruction for Subject 1 (i.e. from 5 mm axial slices) as shown in S2 Fig. Consequently, the model trained using a subject with the thinner slice thickness (5 mm, Subject 1) performed well whereas the model trained with the thicker slice thickness (9 mm, Subject 6) performed poorly for most subjects (Fig 6). In addition, motion between different slices can cause similar aliasing artifacts even for subjects with thinner slices. Although motion was not an obvious problem in this study dataset, it is a major source of suboptimal studies in clinical practice. Thus, subjects with thinner slice thickness and without motion should be selected for training in future studies.

### Limitations

The manual selection of subjects for the training introduces the potential for variability in the results. However, we observed optimal performance of the model when subjects with slice thickness of approximately 5 mm were used. In this study, image processing time for data augmentation took up to two days using a high performing computer cluster. To reduce the training time, the augmented training images have to be generated in advance as these datasets exceeded 2 TB for approaches using 6 subjects. Despite generating augmented images prior to training, our training time was up to two weeks using a single GPU with 32 GB memory. The requirements of a large disk space and a long training time may limit the wide application of this approach. However, these problems may diminish with further improvements in hardware. Similarly, although the training process was time consuming, it did not require human supervision and once the model was trained, testing in new subjects is fast. Due to the limitation of disk space and training time, we did not performed training with more than six subjects. However, we anticipate only limited incremental value for adding more training subjects given the near negligible increase of the Dice coefficient (i.e., 0.003 or 0.33%) when comparing three to six training subjects (Fig 4). Lastly, our segmentation algorithm did not discriminate renal parenchyma from small renal masses (i.e., renal masses were included in the predicted segmentation). Further work is needed to create separate masks of the renal parenchyma and renal masses. In addition, 3D data were required using our approach since 3D augmentation was the key technique to generate labeled training images to achieve few-shot segmentation.

## Conclusion

We demonstrate the feasibility of MR kidney segmentation using deep learning CNN models trained with only a few subjects. Our proposed few-shot CNN approach using 3D augmentation enabled high-quality segmentation of kidney using T1-weighted MR images. The cascaded network and the augmented prediction method further improved the performance of segmentation. Our approach provides a general solution to segmentation in 3D medical imaging when the number of ground truth masks is limited. Further testing of such approaches in other imaging modalities and anatomic locations is necessary.

## Supporting information

S1 Fig Generated example images using 3D rotations. The original data (Green) were rotated sequentially along three different axes with 30 degrees.
(DOCX)

S2 Fig Axial and coronal images from subject 1 and subject 6 in Fig 2. (DOCX)

## References

[1] Gaing B, Sigmund EE, Huang WC, Babb JS, Parikh NS, Stoffel D, et al. Subtype differentiation of renal tumors using voxel-based histogram analysis of intravoxel incoherent motion parameters. Invest Radiol. 2015;50(3):144–52. Epub 2014/11/12. doi: 10.1097/RLI.0000000000000111 . 25387050

[2] de Leon AD, Kapur P, Pedrosa I. Radiomics in Kidney Cancer: MR Imaging. Magn Reson Imaging Clin N Am. 2019;27(1):1–13. Epub 2018/11/24. doi: 10.1016/j.mric.2018.08.005 . 30466904 PMC6554741

[3] LeCun Y, Bengio Y, Hinton G. Deep learning. Nature. 2015;521(7553):436–44. Epub 2015/05/29. doi: 10.1038/nature14539 . 26017442

[4] Ronneberger O, Fischer P, Brox T. U-Net: Convolutional Networks for Biomedical Image Segmentation. arXiv e-prints [Internet]. 2015 May 01, 2015:[arXiv:1505.04597 p.]. https://ui.adsabs.harvard.edu/abs/2015arXiv150504597R.

[5] Akkus Z, Galimzianova A, Hoogi A, Rubin DL, Erickson BJ. Deep Learning for Brain MRI Segmentation: State of the Art and Future Directions. J Digit Imaging. 2017;30(4):449–59. Epub 2017/06/04. doi: 10.1007/s10278-017-9983-4 . 28577131 PMC5537095

[6] Kline TL, Korfiatis P, Edwards ME, Blais JD, Czerwiec FS, Harris PC, et al. Performance of an Artificial Multi-observer Deep Neural Network for Fully Automated Segmentation of Polycystic Kidneys. J Digit Imaging. 2017;30(4):442–8. Epub 2017/05/28. doi: 10.1007/s10278-017-9978-1 . 28550374 PMC5537093

[7] Zhao A, Balakrishnan G, Durand F, Guttag JV, Dalca AV. Data augmentation using learned transformations for one-shot medical image segmentation. 2019 Ieee/Cvf Conference on Computer Vision and Pattern Recognition (Cvpr 2019). 2019:8535–45.

[8] Lei T, Wang R, Wan Y, Zhang B, Meng H, Nandi AK. Medical Image Segmentation Using Deep Learning: A Survey. arXiv e-prints. 2020:arXiv:2009.13120.

[9] Xu C, Howey J, Ohorodnyk P, Roth M, Zhang H, Li S. Segmentation and quantification of infarction without contrast agents via spatiotemporal generative adversarial learning. Med Image Anal. 2020;59:101568. Epub 2019/10/18. doi: 10.1016/j.media.2019.101568 . 31622838

[10] Bobo MF, Bao S, Huo Y, Yao Y, Virostko J, Plassard AJ, et al. Fully Convolutional Neural Networks Improve Abdominal Organ Segmentation. Proc SPIE Int Soc Opt Eng. 2018;10574. Epub 2018/06/12. doi: 10.1117/12.2293751 . 29887665 PMC5992909

[11] Li FF, Fergus R, Perona P. One-shot learning of object categories. Ieee T Pattern Anal. 2006;28(4):594–611. doi: 10.1109/Tpami.2006.79 16566508

[12] Zhou ZH. A brief introduction to weakly supervised learning. Natl Sci Rev. 2018;5(1):44–53. doi: 10.1093/nsr/nwx106

[13] Wang Y, Yao Q, Kwok J, Ni LM. Generalizing from a Few Examples: A Survey on Few-Shot Learning2019 April 01, 2019:[arXiv:1904.05046 p.]. https://ui.adsabs.harvard.edu/abs/2019arXiv190405046W.

[14] Rakelly K, Shelhamer E, Darrell T, Efros AA, Levine S. Few-Shot Segmentation Propagation with Guided Networks2018 May 01, 2018:[arXiv:1806.07373 p.]. https://ui.adsabs.harvard.edu/abs/2018arXiv180607373R.

[15] Dong N, Xing EP. Few-Shot Semantic Segmentation with Prototype Learning. BMVC; NewCastle, UK 2018. p. 4.

[16] Caelles S, Maninis KK, Pont-Tuset J, Leal-Taixe L, Cremers D, Van Gool L. One-Shot Video Object Segmentation. Proc Cvpr Ieee. 2017:5320–9. doi: 10.1109/Cvpr.2017.565 29994298

[17] Guha Roy A, Siddiqui S, Pölsterl S, Navab N, Wachinger C. ’Squeeze & Excite’ Guided Few-Shot Segmentation of Volumetric Images. arXiv e-prints. 2019:arXiv:1902.01314. 10.1016/j.media.2019.101587 31630012

[18] Wang K, Hao Liew J, Zou Y, Zhou D, Feng J. PANet: Few-Shot Image Semantic Segmentation with Prototype Alignment. arXiv e-prints. 2019:arXiv:1908.06391.

[19] Zhang X, Wei Y, Yang Y, Huang TS. SG-One: Similarity Guidance Network for One-Shot Semantic Segmentation. IEEE Trans Cybern. 2020;50(9):3855–65. Epub 2020/06/05. doi: 10.1109/TCYB.2020.2992433 . 32497014

[20] Feng R, Zheng X, Gao T, Chen J, Wang W, Chen DZ, et al. Interactive Few-Shot Learning: Limited Supervision, Better Medical Image Segmentation. IEEE Trans Med Imaging. 2021;40(10):2575–88. Epub 2021/02/20. doi: 10.1109/TMI.2021.3060551 . 33606628

[21] Valverde S, Salem M, Cabezas M, Pareto D, Vilanova JC, Ramio-Torrenta L, et al. One-shot domain adaptation in multiple sclerosis lesion segmentation using convolutional neural networks. Neuroimage Clin. 2019;21:101638. Epub 2018/12/18. doi: 10.1016/j.nicl.2018.101638 . 30555005 PMC6413299

[22] Chen X, Lian C, Wang L, Deng H, Fung SH, Nie D, et al. One-Shot Generative Adversarial Learning for MRI Segmentation of Craniomaxillofacial Bony Structures. IEEE Trans Med Imaging. 2020;39(3):787–96. Epub 2019/08/20. doi: 10.1109/TMI.2019.2935409 . 31425025 PMC7219540

[23] Chen X, Yang Y, Wang S, Wu H, Tang J, Zhao J, et al. Ship Type Recognition via a Coarse-to-Fine Cascaded Convolution Neural Network. Journal of Navigation. 2020;73(4):813–32. Epub 2020/02/28. doi: 10.1017/S0373463319000900

[24] Liu Z-T, Li S-H, Wu M, Cao W-H, Hao M, Xian L-B. Eye localization based on weight binarization cascade convolution neural network. Neurocomputing. 2020;378:45–53. doi: 10.1016/j.neucom.2019.10.048

[25] Budak U, Guo Y, Tanyildizi E, Sengur A. Cascaded deep convolutional encoder-decoder neural networks for efficient liver tumor segmentation. Med Hypotheses. 2020;134. ARTN 109431 doi: 10.1016/j.mehy.2019.109431 31669758

[26] Dey R, Hong Y. Hybrid Cascaded Neural Network for Liver Lesion Segmentation. I S Biomed Imaging. 2020:1173–7.

[27] Sobhaninia Z, Rezaei S, Karimi N, Emami A, Samavi S. Brain Tumor Segmentation by Cascaded Deep Neural Networks Using Multiple Image Scales2020 February 01, 2020:[arXiv:2002.01975 p.]. https://ui.adsabs.harvard.edu/abs/2020arXiv200201975S.

[28] Dou Q, Chen H, Yu LQ, Zhao L, Qin J, Wang DF, et al. Automatic Detection of Cerebral Microbleeds From MR Images via 3D Convolutional Neural Networks. Ieee T Med Imaging. 2016;35(5):1182–95. doi: 10.1109/Tmi.2016.2528129 26886975

[29] Emre Kavur A, Sinem Gezer N, Barıș M, Aslan S, Conze P-H, Groza V, et al. CHAOS Challenge—Combined (CT-MR) Healthy Abdominal Organ Segmentation2020 January 01, 2020:[arXiv:2001.06535 p.]. https://ui.adsabs.harvard.edu/abs/2020arXiv200106535E.

[30] Tustison NJ, Avants BB, Cook PA, Zheng Y, Egan A, Yushkevich PA, et al. N4ITK: improved N3 bias correction. IEEE Trans Med Imaging. 2010;29(6):1310–20. Epub 2010/04/10. doi: 10.1109/TMI.2010.2046908 . 20378467 PMC3071855

[31] He K, Zhang X, Ren S, Sun J. Deep Residual Learning for Image Recognition2015 December 01, 2015:[arXiv:1512.03385 p.]. https://ui.adsabs.harvard.edu/abs/2015arXiv151203385H.

[32] Xie S, Tu Z. Holistically-Nested Edge Detection2015 April 01, 2015:[arXiv:1504.06375 p.]. https://ui.adsabs.harvard.edu/abs/2015arXiv150406375X.

[33] Jadon S. A survey of loss functions for semantic segmentation2020 June 01, 2020:[arXiv:2006.14822 p.]. https://ui.adsabs.harvard.edu/abs/2020arXiv200614822J.

[34] Cui H, Wei D, Ma K, Gu S, Zheng Y. A Unified Framework for Generalized Low-Shot Medical Image Segmentation With Scarce Data. IEEE Trans Med Imaging. 2021;40(10):2656–71. Epub 2020/12/19. doi: 10.1109/TMI.2020.3045775 . 33338014

[35] Cubuk ED, Zoph B, Mane D, Vasudevan V, Le QV. AutoAugment: Learning Augmentation Policies from Data2018 May 01, 2018:[arXiv:1805.09501 p.]. https://ui.adsabs.harvard.edu/abs/2018arXiv180509501C.

[36] Minaee S, Boykov Y, Porikli F, Plaza A, Kehtarnavaz N, Terzopoulos D. Image Segmentation Using Deep Learning: A Survey2020 January 01, 2020:[arXiv:2001.05566 p.]. https://ui.adsabs.harvard.edu/abs/2020arXiv200105566M. 10.1109/TPAMI.2021.3059968 33596172

[37] Aganj I, Yeo BT, Sabuncu MR, Fischl B. On removing interpolation and resampling artifacts in rigid image registration. IEEE Trans Image Process. 2013;22(2):816–27. Epub 2012/10/19. doi: 10.1109/TIP.2012.2224356 . 23076044 PMC3694571

[38] Park SK, Schowengerdt RA. Image sampling, reconstruction, and the effect of sample-scene phasing. Appl Opt. 1982;21(17):3142–51. Epub 1982/09/01. doi: 10.1364/AO.21.003142 . 20396192

[39] Parker J, Kenyon RV, Troxel DE. Comparison of interpolating methods for image resampling. IEEE Trans Med Imaging. 1983;2(1):31–9. Epub 1983/01/01. doi: 10.1109/TMI.1983.4307610 . 18234586

