# Supplementary material for: Deep learning kidney segmentation with very limited training data using a cascaded convolution neural network
Source: PLoS One. 2022 May 9;17(5):e0267753. doi: 10.1371/journal.pone.0267753 (PMC9084530; doi:10.1371/journal.pone.0267753)
Supplement: S1 Fig — The original data (Green) were rotated sequentially along three different axes with 30 degrees. (DOCX) [file pone.0267753.s001.docx]

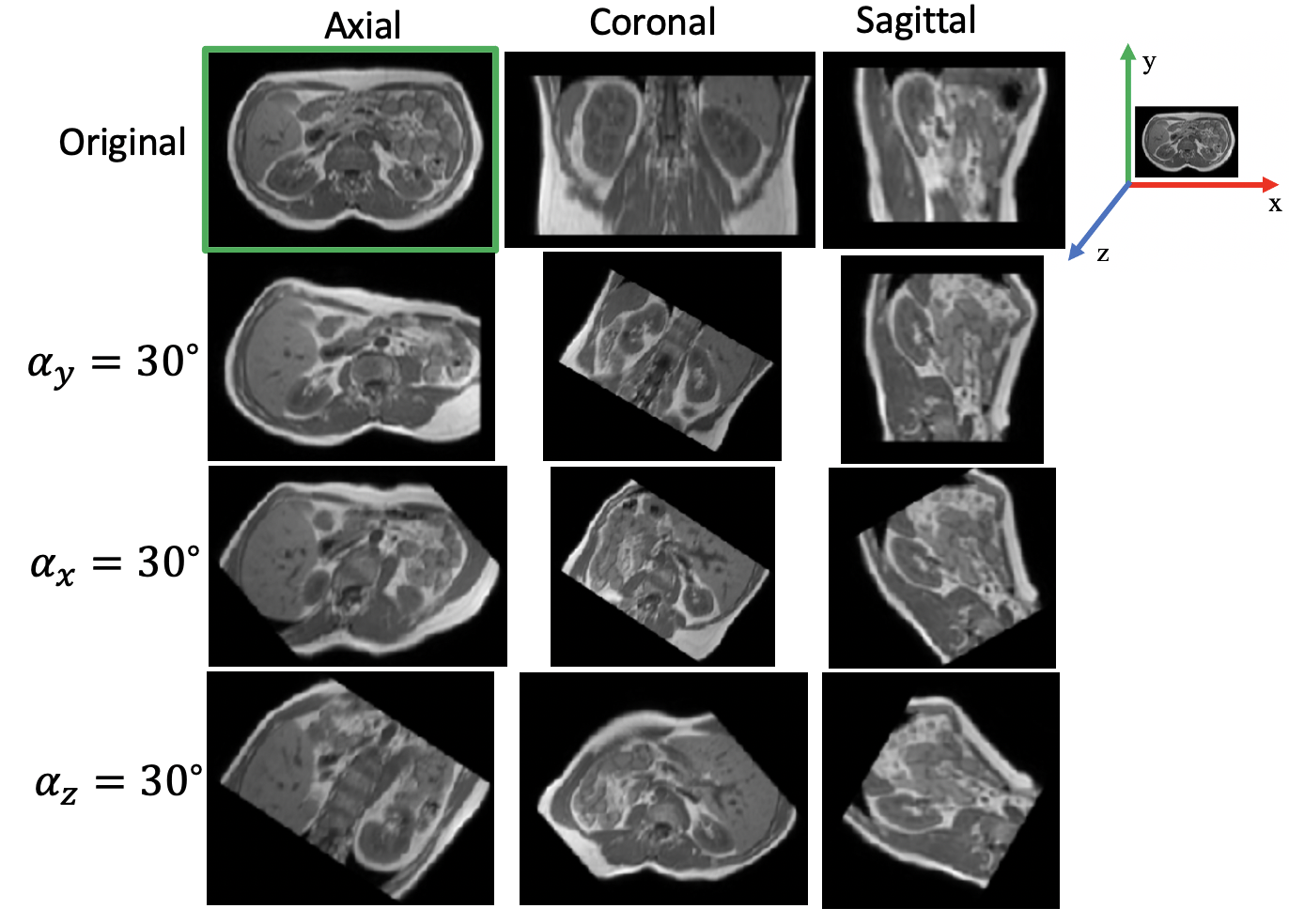


**S1 Fig. Generated example images using 3D rotations.** The original data (Green) were rotated sequentially along three different axes with 30 degrees.
