# Supplementary figures and images for: Deep learning kidney segmentation with very limited training data using a cascaded convolution neural network
Source: PLoS One. 2022 May 9;17(5):e0267753. doi: 10.1371/journal.pone.0267753 (PMC9084530; doi:10.1371/journal.pone.0267753)

**
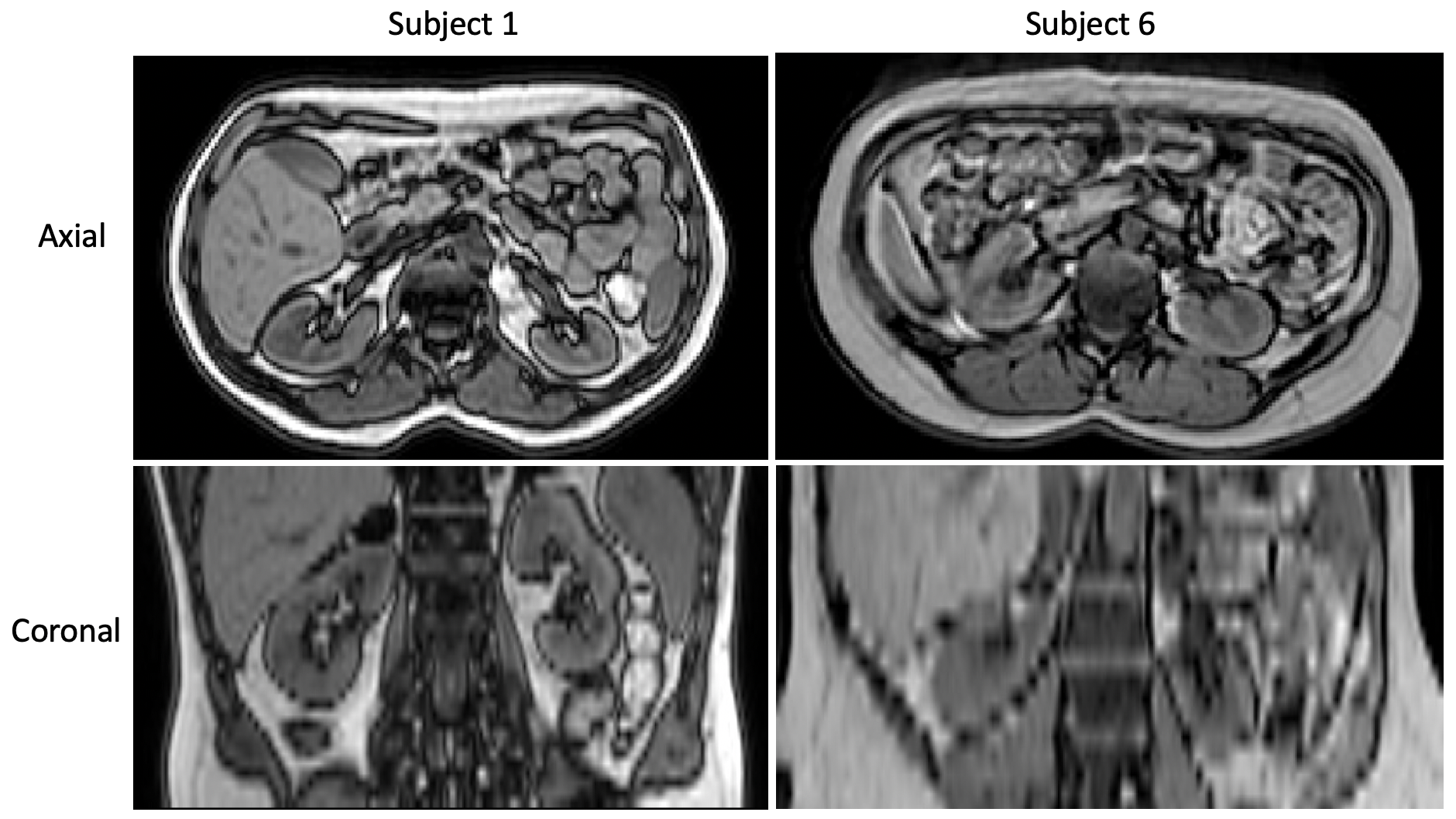
**

**S2 Fig. Axial and coronal images from subject1 and subject 6 in Fig 2.**

Supplement: S2 Fig — (DOCX) [file pone.0267753.s002.docx]
